# Report of a Case of Double Dislocation of the Knee-Joints

**Published:** 1892-04

**Authors:** Thomas H. Manley

**Affiliations:** Visiting Surgeon to Harlem Hospital, New York; 302 West Fifty-third Street


Buffalo Medical and Surgical Journal

Vol. XXXI. APRIL, 1892. No. 9.

### Original Communications.

### REPORT OF A CASE OF DOUBLE DISLOCATION OF THE KNEE-JOINTS.

By THOMAS H. MANLEY, M. D.,
Visiting Surgeon to Harlem Hospital, New York.

In the city of Buffalo, Hamilton commenced those serial observations some years ago, which led to the publication of the first work printed in America on Fractures and Dislocations, and the most classical and complete exposition on the subject that had, up to that time, ever been written in the English language. As a matter of reverence to the memory of that great surgeon, and high regard for our brethren in the great western city, I have decided to offer something in the way of a report of a case of dislocation, to the Buffalo Medical and Surgical Journal, which, if it possesses no other value, it will deserve some merit, at least, in being, in many particulars, quite unique.

Besides reporting this singularly interesting case, I will take this opportunity to call the attention of physicians to a few features in connection with dislocations in general.

The subject of this report was:

A man thirty-three years old; medium height and build, who commonly enjoyed good health; he never had any serious malady, nor sustained severe injury before, being employed in a cabinet factory, running a planing machine. On the morning of October 2, 1891, while endeavoring to put his machine in motion, he was caught by the belt and carried with violent force over the shaft and pulley. The shaft was twenty-five feet from the floor, but the space between the over-hanging beams and the outer belt-surface of the pulley above was scarcely two feet. As his body was carried over the shaft, he struck the floor beams with terrible force, and was projected fifteen feet in a direction diagonally to the revolving shaft. It was at first supposed by his fellow-workers that he had been instantly killed, as he was completely motionless; bleeding from the mouth, nose and wounds about the face. After a few minutes, however, he commenced to audibly gasp, when the ambulance was summoned.

*Ambulance History.*—Dr. Arch. Dixon, of the Harlem hospital house-staff, responded. His report of the case says: "That when the ambulance arrived, about twenty minutes after the accident, I saw the patient lying on his back in a state of profound collapse, the pulse being thready, very rapid and intermittent. The breathing was very shallow and sighing. His extremities were cold. There was a moderate drooling of blood from the mouth, while there was a free oozing from the right nostril. The bleeding from the mouth was attributable to slight abrasions, as there was no hemorrhagic expectoration from the lungs, nor was any blood raised in vomiting. He was perfectly conscious. There was no evidence of serious spinal injury, as there was no paralysis. Giving him whiskey by the mouth and digitaline by hypodermic injection, after a little while he so far recuperated that it was deemed safe to slightly shift his position, in order to make a superficial examination. As he lay flat on his back, it was evident, from the position of the toes, that there was joint or bone injury of the left leg, for the foot fell outwards and rested on its side, and the knee was slightly bent. On removing his coat it was found that he had a compound fracture of the right humerus close to the surgical neck, the inferior fragment protruding through the skin. On the same side of the body, at about the center of their shafts, the fifth, sixth, seventh and eighth ribs were fractured. After having had his trousers removed, it was evident that both knee-joints were dislocated. On the right side the condyles of the femur were displaced downward and forward, the head of the tibia rising high into the popliteal space, slightly adducted and rotated outward. There was no ecchymosis or extravasation into the cellular tissues. On the left side the dislocation was the reverse of the right with respect to the head of the femur, as here the condyles were displaced posterior to the head of the tibia, crowding down into the loose tissues of the popliteal space. The head of the tibia, owing to the manner in which it had risen into the capsule of the joint, had the articular surface of the patella lying flat across its horizontal surface. I was able to easily restore the joint surfaces to their proper relations on the right side, but the left dislocation required considerable manipulation and force before we succeeded. His knees were now, with the fractured arm, adjusted in splints, when he was placed in the ambulance and brought to the hospital."

*Admission to Hospital.*—Two hours after he was admitted into the male surgical ward I saw him. At this time he was yet in deep shock. His pulse was so feeble, and his integuments so blanched, that it seemed to me that he must be suffering from internal hemorrhage. The surface temperature was low, and his pulse was very feeble. His intellectual faculties were undimmed, and he answered questions, though in feeble accents. He said he had no pain anywhere; that he had a numb, tingling feeling in the soles of both feet; that he felt cold, very weak, and thought he was dying. At this juncture all external hemorrhage had ceased. Regarding the injuries of a mortal character, he was not disturbed; but the nurses, nevertheless, were directed not to relax their efforts in endeavoring to arouse the ebbing vitals, and establish reaction. From his general condition it did not seem that he could survive more than a few hours. But he consumed hot whiskey freely, and, after having been covered on every side by artificial heat, after midnight he commenced to react, so that when I saw him again early the following morning, he was fairly restored, when a more critical examination of his injuries was made, in order to learn of their precise character, and institute such remedial measures as might be deemed most appropriate.

Before proceeding with a description of his condition, it may be well, for a moment, to consider the nature and extent of physical force brought into action in this case. We see, at the outset, that the violence sustained was inflicted mainly in three ways. First, he was instantaneously jerked from his feet with such suddenness as we might expect would wring a bone from its socket, or crush in a shaft. Secondly, he was shot through a narrow space, between the pulley and the rafters, with such force as one might suppose would scarcely leave a whole bone in his body—long bone. How he was carried through this narrow, irregular space without having a limb torn off, or an internal organ ruptured, is a mystery. Third, he was thrown with great force a considerable distance, striking on his right side against a machine, which was not, fortunately, in motion. So that we see that he was subjected to a triplicate force of great relative intensity, and hence, from a description of the manner in which the injuries were sustained, we might be prepared to find serious and multiple lesions.

On removing the bandages from the lower limbs, it was evident that the circulation below the femoro-tibial articulation was almost or completely arrested, as both legs and feet were cool, the left more than the right. No throb could be felt in either the posterior tibial, or dorsalis pedis, on either side. Besides, on surface pressure, the emptied capillaries failed to refill again. The ligaments, muscles, and tendons on the right side were so badly torn and stretched that it was found that if the heads of the bones were not fixed in contact with each other by some sort of a mechanical adjustment, the foot would fall on its side, and the tibial head would rotate outward, the patella slipping inward and resting on its inner margin. The muscular and ligamentous disorganization on the left side was apparently not so extensive; for in their articulation the luxated bones remained in position without any tendency to spontaneous displacement.

At this time there was an extensive hemorrhagic effusion into the capsules of both knees, and ecchymotic straining of the integuments of the thighs, on their outer aspects from the major trochanter downward. Both limbs were now comfortably readjusted and artificial warmth maintained, with a view of preserving vitality and favoring the establishment of the circulation through the collateral channels. The principal articular and long sural arteries were now depended on to maintain vitality, for it was at this time quite clear that both popliteal trunks had suffered irreparable damage, and that all hope of saving the limbs depended on our ability to nourish the parts below by way of the subsidiary vessels. And this, it would seem, we would have accomplished had we the *vis a tergo*, the strong heart stroke, to pump the blood through the now narrowed portals. But our unfortunate man had his right thoracic chamber crushed in, the shivered costal shafts having evidently been driven deeply into the right pulmonary parenchyma, as the lung was now crowded far up under the superior ribs, and the pleura was filled with blood. Added to this there was an extensive emphysema into the cellular tissues of the lumbar, liver, and costo-spinal regions. The fracture of the humerus was of the oblique variety, with a tendency to adduction and rotation inward of the superior fragment by the infra- and supra-spinatus muscles. By firm padding of the axilla and forced flexion inward of the arm, this was fairly overcome, and the arm was fixed in a comfortable adjustment.

It is particularly interesting to note, in this connection, that notwithstanding the terrible injuries inflicted, the concussion, stretching, squeezing, and violent bruising which was sustained, the spinal-marrow wholly escaped, as did also the cerebral ganglia. He had perfect rectal and vesical control. There was no evidence of injury to any of the solid or hollow viscera of the abdomen.

Singular to say, in spite of the great force necessary to dissunder the powerful structures at both knee-joints, and cause the other serious injuries noted, this man rallied well, and had it not been for the consecutive events arising from disorganization of the popliteal vessels, he would have undoubtedly made a good recovery.

On the second day after admission, his general condition was very good. His temperature was under 100, and the pulse was 85, in the morning. There was but slight thirst. He had no chills, and there had been no vomiting. His arm was painful at the seat of fracture, and now that reaction was well established, there was considerable swelling in the loose connective tissues, external to the synovial investments of the knee-joints, which extended along the muscle planes to the groins. These areas were extremely sore and tender. At this time the left leg and foot presented an ominous aspect. From the knee down, the limb had a livid, bloated appearance, and there seemed an entire absence of circulation in the peripheral capillaries. The parts supplied mainly by the short saphenous, the muscular, cutaneous and anterior tibial nerves, were completely paralyzed. On the sole of the foot, along the parts vitalized by the internal and deep plantar branches, from the posterior tibial, the reflexes were yet intact, and normal sensation was but little dulled.

From the symptoms and signs, the rapid increase in volume in a limb almost devoid of circulation, it was rather suggestive of chemical changes, and the commencement of that process through which nature demands the return of the original elements, of which a part is physically constituted—of decomposition. Certainly, we might witness a rapid increase in the dimensions of a part by pathological processes, by cellular hyperplasia, edema, hemorrhage, etc., yet these are *vital* processes, and are only possible when the circulation is intact. As his general condition was now good, I ventured to recommend immediate amputation above the knee, but the patient demurred, and the family physician advised against it, at this stage. In the meantime, the limbs, from the toe nails up to above the knees, were kept thoroughly cleansed and swathed in antiseptic gauze. Gangrenous processes, however, went on, about the same as they always do under these circumstances.

On the morning of the third day unmistakable evidences of the total death of everything below the knee was evident, and gangrenous changes were rapidly advancing towards the trunk, so that it now became a question of disarticulation at the hip-joint. The cadaverous odor of the decomposing corporeal elements was widely diffused throughout the ward. The limb itself had become, in patches, charred, blackened and shrunken, the epithelium peeling off on the slightest rub, and now everywhere, below the knee, sensation was totally lost. As yet there was no evidence of pollution of the circulation through the absorption of the lethal elements of sphacelus.

On the afternoon of this day, after his consent had been reluctantly given, I amputated through the lower third of the femur. He rallied well from the operation, and, when the stump was dressed, two days after amputation, it was seen that there had been primary union along the entire seam of incision.

On the tenth day after admission, and the seventh after amputation, we were appalled to find evidence of peripheral gangrene, appearing in the right leg in brown, irregularly-formed plaques. Those patches looked as though the aqueous, albuminous, and fatty elements had been withdrawn from the involved integument, which gave it the appearance of the dried raw-hide of a drum-head. It was clear that while the circulation was not wholly shut off in this limb in the deeper structures, yet it was unmistakable that the surface structures were not amply nourished.

He positively refused to submit to an amputation of this limb, and, I think, wisely, for the infliction of another high amputation mutilation, on his already shattered anatomy, would, no doubt, have been more than he could endure. In this limb, as in its fellow, after the first appearance of gangrene, it rapidly ran into sphacelus, and reached the lower abdomen within forty-eight hours. He died on the nineteenth day after admission, from septic pneumonia.

For many important reasons it might seem proper that a case of this description should be reported in full. First, for its uniqueness. I can find no report of a case of *double* dislocation of the knees by any American author. Our principal authors on Dislocations, Hamilton[1] and Stimpson[2], do not tell us that they ever saw a case. Hamilton practically passes over the subject in silence, though Stimpson, in his book, citing from the French, mainly, mentions several cases which he was able to find. Among them there was one which occurred in the hospital service of Le Dentu, of La Pitié, in Paris. His man, like mine, was caught in a belt, dragged over a shaft, and sustained a double dislocation of both knee-joints, with the condyles of the femur occupying reversed positions, as in this case. It appears that this patient recovered with paralysis of one limb, and gangrene of the tendo-Achilles in the other.

*Pathological Examination of the Amputated Limb.*—As the incision into the soft parts, in this amputation of the limb, was car-

1. Hamilton, Fractures and Dislocations.
2. Stimpson, Fractures and Dislocations.

ried through a point above the joint, an opportunity was permitted us to examine the entire articulation and the morbid anatomy consequent on the injury. The tegumentary envelope had a bluish-black color throughout its entire extent. Between the cellular fascia and the skin, there was a dark, watery accumulation, of a foul odor. On dividing the thick fibrous aponeurosis of the leg, a large quantity of partly disorganized and coagulated blood escaped. This was very abundant in the popliteal space. The muscles and fibro-cellular elements were all deeply blood-stained. Between the muscle-sheaths in the direction of the long bones, gaseous decomposition was very general, so that on separating the muscles, there was a crepitating sound emitted from the inflated tissues.

Coming down to the great blood-trunks in the popliteal space, the popliteal artery was found completely thrombosed, from about half an inch below Hunter's canal, the coagulum extending into the anterior tibial and the first division of the posterior tibial. The peroneal, from this point downward, was pervious and empty. The internal coat of the artery, under the seat of the thrombus, was congested, thickened, and in places detached from the muscular coat; besides, there was a thin layer of organized lymph, easily detachable from the serosa. The cellular tunic of the vessel was greatly thickened, and, on microscopical examination, showed evidence of having sustained an extensive hemorrhagic extravasation, probably from the vasa-vasorum. The popliteal nerve, throughout its medulla, was softened, and in patches stained by foci of ecchymosis. Its neurilemma, however, was not torn.

The popliteal vein, at the point immediately below that at which the sural vessels enter, had been ruptured; but adhesive inflammation, in the over-lying tissues, had so approximated its divided walls, that there certainly was no leakage through it at the time of death. Its contents were of a black, tarry quality. The popliteal muscle was nearly completely torn off from its attachment into the outer condyle of the femur, and both heads of the gastrocnemii at their attachments showed evidences of extensive bruising and laceration.

Permission was refused us to dissect the other limb after death.

The report of the hospital pathologist on the complete microscopical examination is not received, or it would have been incorporated.

The untoward consequences to the extremities following these two luxations, forcibly present to us the direful results which may occur after a dislocation in any of the articulations, particularly those of the extremities. This we can easily comprehend in the case of the knee, which, when dislocated, is acted upon by great force and compound motion; for, to completely effect surface displacement here, we must have combined a violent bending, twisting, and extension force acting simultaneously. When this force is of sufficient velocity and intensity to dislodge the articular heads of bones, held in position by so many powerful tendons, muscles, and ligaments as we find at the knee-joint, we may always not only expect great shock from commotion of the whole body, but likewise extensive implication of the large nerve-trunks and blood-vessels, which, at the knee-joint particularly, are firmly held in position by an expansion of the fascia lata and tendons; and, besides, are in this situation the more immovably fixed by their divisions and subdivisions, which hold them down like so many roots, and make their abrupt displacement, without laceration or destructive contusion, scarcely possible. So that although we may, as in this case, succeed in prompt replacement of the luxated bones, yet the damage done to vital structures remains. Accordingly the great danger to life or limb is in those cases always of a consecutive character when no internal organ is implicated.

Stimpson,[1] in his unrivaled work on the subject of Dislocations, says: "That those complete dislocations at the knee derive their greatest importance from the grave consequences which so often arise from lesions of the blood-vessels, gangrene, paralysis," etc.

The great French surgeon,[2] Velpeau, was no less emphatic in his lessons on dislocations in general.

Although the quality of force is not always the same in dislocations as in fractures, yet, as a general rule, it may be said, a greater amount of force is necessary to dislocate a joint than to fracture a single shaft of bone. In these joints, the composition of which is principally ligamentous and tendinous, as the knee or ankle, a complete luxation is almost invariably followed by great limitation in motion and impairment of strength, to say nothing of the atrophy, wasting, or palsy, which may involve the parts beyond. The most common dislocation is that at the shoulder-joint. It is also the most harmless and easily remedied; yet I have seen as many as ten shoulder-joint dislocations which were positively irreducible.

1. Stimpson on Dislocations, page 317.
2. Velpeau, Leçon Sur Les Dislocations.

I once saw a surgeon in Bellevue hospital, in the presence of the late Frank Hamilton, fracture the humerus in attempting to replace its head. On another occasion, at Charity Hospital, a famous surgeon, on joint surgery particularly, produced a large fleshy woman who had a luxated shoulder, in order to demonstrate how easily (?) those things were remedied when one only understood them. But after trying every conceivable method, and exhausting his own herculean strength, he utterly failed.

In the winter of 1884 I had two cases of irreducible shoulder-joint dislocations at the Work-house hospital, Blackwell's Island. Some of the visiting staff had seen them, and I brought over Dr. J. W. S. Gouley to assist me, but nothing could be done; the bones remained unreduced. Having seen Schrœder's method for reducing dislocated shoulders, its simplicity attracted me, and a physician having invited me in to reduce a dislocated shoulder, I employed this method. I had scarcely commenced to bring into action the full leverage which the plan affords, when the humeral shaft broke like a pipe stem. Prof. Dennis exhibited a case before the surgical section of the New York Academy of Medicine two years ago, of a boy who, after a dislocated shoulder, lost the use of his arm, which had to be amputated later. The great desideratum in the treatment of dislocations is to employ such force and direct it in such a manner as will favor the return of the displaced bones, without doing irreparable damage to the nerves, blood-vessels, or adjacent muscles producing a fracture; otherwise it is much better to leave a limb unreduced in an obstinate case than to take chances on the employment of great force.

302 West Fifty-third Street.

---

The Prevalent Fashion of Self-Medication.—The writer has had frequent opportunities to observe the growing tendency, at the present day, among those wholly unacquainted with the action of medicines, to the self-administration of the most powerful remedies. Surely, never before these advanced days, has it been customary for men and women to prescribe for their own use, without consultation with medical men as to the properties of the case, medicines which, in large doses, are dangerous, even to a toxical degree. Should there not be some restriction upon the sale by pharmacists of many of these potent agents, not in the interest of the medical profession, but in that of the venturesome experimenters themselves, whose lives are often jeopardized by such injudicious self-medication?—*Editorial in College and Clinical Record.*

